# Transcriptome Profile Analysis of *Arabidopsis* Reveals the Drought Stress-Induced Long Non-coding RNAs Associated With Photosynthesis, Chlorophyll Synthesis, Fatty Acid Synthesis and Degradation

**DOI:** 10.3389/fpls.2021.643182

**Published:** 2021-05-25

**Authors:** Kang Chen, Yang Huang, Chunni Liu, Yu Liang, Maoteng Li

**Affiliations:** ^1^Department of Biotechnology, College of Life Science and Technology, Huazhong University of Science and Technology, Wuhan, China; ^2^Key Laboratory of Ecology of Rare and Endangered Species and Environmental Protection, College of Life Science, Guangxi Normal University, Guilin, China; ^3^School of Mechanical and Electrical Engineering, Guilin University of Electronic Technology, Guilin, China

**Keywords:** fatty acid synthesis, chlorophyll synthesis, lncRNAs, *VOC*, *LEA3*

## Abstract

Long non-coding RNAs (lncRNAs) play an important role in the response of plants to drought stress. The previous studies have reported that overexpression of *LEA3* and *VOC* could enhance drought tolerance and improve the oil content in *Brassica napus* and *Arabidopsis thaliana*, and most of the efforts have been invested in the gene function analysis, there is little understanding of how genes that involved in these important pathways are regulated. In the present study, the transcriptomic results of *LEA3* and *VOC* over-expressed (OE) lines were compared with the RNAi lines, mutant lines and control lines under long-term and short-term drought treatment, a series of differentially expressed lncRNAs were identified, and their regulation patterns in mRNA were also investigated in above mentioned materials. The regulation of the target genes of differentially expressed lncRNAs on plant biological functions was studied. It was revealed that the mutant lines had less drought-response related lncRNAs than that of the OE lines. Functional analysis demonstrated that multiple genes were involved in the carbon-fixing and chlorophyll metabolism, such as *CDR1*, *CHLM*, and *CH1*, were regulated by the upregulated lncRNA in OE lines. In LEA-OE, *AT4G13180* that promotes the fatty acid synthesis was regulated by five lncRNAs that were upregulated under both long-term and short-term drought treatments. The key genes, including of *SHM1, GOX2*, and *GS2*, in the methylglyoxal synthesis pathway were all regulated by a number of down-regulated lncRNAs in OE lines, thereby reducing the content of such harmful compounds produced under stress in plants. This study identified a series of lncRNAs related to the pathways that affect photosynthesis, chlorophyll synthesis, fatty acid synthesis, degradation, and other important effects on drought resistance and oil content. The present study provided a series of lncRNAs for further improvement of crop varieties, especially drought resistant and oil content traits.

## Background

Drought is one of the most common abiotic stresses that plants face, and which is a leading cause of crop failure globally (Lesk et al., 2016). In recent years, various studies have focussed on the biology of drought tolerance and laid some foundations on the mechanism underlying the drought resistance of plants. Some important genes and pathways that related to drought stress have been identified, such as the dehydration response element binding proteins (DREBs) (Agarwal et al., 2006) and abscisic acid signal transduction system (Cai et al., 2017). Consequently, these multiple genes involved in a complex regulatory networks related to drought tolerance have posed challenges in the selection of drought tolerance traits in plant breeding. *Late embryogenesis abundant group 3* (*LEA3*) genes are part of a category that could function in the protection of membranes and proteins. *Vicinal oxygen chelate* (*VOC*) proteins are members of an enzyme superfamily with a common mechanistic attribute enabled by conserved active site residues. In our previous study, we found that overexpression of *LEA3* and *VOC* genes could increase the drought resistance and oil content in *Arabidopsis* and *B. napus* (Liang et al., 2019).

Long non-coding RNA (lncRNA) refers to the RNA that is longer than 200 nucleotides and is not capable of translation into protein. Some IncRNAs can take part in the regulation of response to abiotic stresses with genes (Liu et al., 2013). A large number of lncRNAs have been identified in multiple species with the rapid development of high-throughput sequencing technology, and some of them could be classified as polyadenylic acid and non-polyadenylic acid (Liu et al., 2012, 2013). The lncRNA plays an important role in response to abiotic stress in plants. Previous studies have shown that the regulatory mechanism of lncRNA is very complex. For example, lncRNA could regulate the expression of protein-coding genes in *cis* or *trans* format and plays a regulatory role in modification of histones (Wang et al., 2011; Kung et al., 2013). In cassava, 124 lncRNAs under drought treatment that exhibited significant effects on carbohydrate metabolism, the Calvin cycle, light response, and light signalling, have been identified (Ding et al., 2019). Xiao et al. (2019) have identified two lncRNAs (LNC_001148 and LNC_000160) that related to drought resistance of tetraploid cassava, which mediate drought tolerance by regulating the stomatal density (Xiao et al., 2019). Drought-responsive lncRNA has been discovered to play a role as a transcription regulator in tomato, and it has a certain regulatory effect on tolerance-related genes such as stimuli, signalling, and response to transporter activity (Eom et al., 2019). A recent study in *Arabidopsis thaliana* also identified an important drought-induced lncRNA (lncRNA-DRIR) which had the key role in regulating plant tolerance to drought and salt stress (Qin et al., 2017). In addition, a large number of lncRNAs related to stress have been identified in corn, grape, Tibetan wild barley and pepper (Fei et al., 2019; Pang et al., 2019; Qiu et al., 2019; Wang et al., 2019).

*B. napus* (AACC, 2n = 38) is an allopolyploid species with a triplicated genome structure and many duplicated genes (Chalhoub et al., 2014). Rapeseed is the third largest oil seed crop in the world, and many areas where it is planted are affected by drought. Thus, it is important to further study the exact roles of genes involved in oil accumulation and response to drought stress conditions.

*LEA* genes are part of a category that could function in the protection of membranes and proteins. Some studies have suggested that LEA-type proteins could act as water-binding molecules, playing important roles in macromolecule and membrane stabilisation and in ion sequestration (Wang et al., 2003; Chakrabortee et al., 2012). These structural characteristics are related to the prediction of their function in response to desiccation stress (Chakrabortee et al., 2007; Candat et al., 2014). VOC proteins are members of an enzyme superfamily with a common mechanistic attribute enabled by conserved active site residues. Glyoxalase I (GLYI) is a major member of the VOC family. It is a metalloenzyme that participates in the glyoxalase system, which has been reported to be a major pathway for the detoxification of methylglyoxal (MG) in living organisms. GLYI can use one molecule of glutathione (GSH) to convert MG to S-D-lactoylglutathione and functions in abiotic stress response (Singla-Pareek et al., 2003; Mustafiz et al., 2014).

In our previous study, it was revealed that the LEA and VOC proteins were highly expressed in *B. napus* plants that had high oil content by proteomics analysis (Gan et al., 2013). We also found that most of the *BnLEA* and *BnVOC* genes had higher expression levels under drought stress in *B. napus* (Liang et al., 2016, 2017). The overexpression (OE) of different copies of the drought response genes *LEA3* and *VOC* enhanced both drought tolerance and oil content in *B. napus* and *Arabidopsis*. In contrast, oil content and drought tolerance were decreased in the *AtLEA3* mutant (*atlea3*) and AtVOC-RNAi of *Arabidopsis* and in both BnLEA-RNAi and BnVOC-RNAi *B. napus* RNAi lines. Hybrids between two lines with increased or reduced expression (LEA3-OE with VOC-OE, *atlea3* with AtVOC-RNAi) showed corresponding stronger trends in drought tolerance (Liang et al., 2019).

All these results showed that *LEA3* and *VOC* genes can play an important role in drought response (not only AtLEA and AtVOC genes, but also BnLEA and BnVOC genes). And different transgenic plants lines which can be used for further study, including the OE lines, RNAi lines, knock-out mutants and hybrids. In the previous study, we have demonstrated the differential expression patterns of protein-coding genes and their corresponding effects in important pathways (Liang et al., 2019). In the present study, the expression pattern of lncRNA in each sample, and their effect on the regulation of target genes on plant drought resistance and key pathways related to lipid synthesis were analysed in order to investigate the role of LEA and VOC genes in long-term and short-term drought stress. 2744 known lncRNAs and 1113 new lncRNAs in 56 samples were identified, and a large number of genes, especially in the metabolism of carbon and chlorophyll, that are regulated by lncRNA were observed. Among which, some single genes are regulated by multiple lncRNAs, such as CRD1 are trans-regulated by AT1G07993, AT1G09513, AT5G15845, AT2G08665, AT1G05207, AT3G08795, AT5G02095, and AT2G23672. The current study revealed that lncRNA plays an important role in the regulation of abiotic stress and enhances oil accumulation in *Arabidopsis*.

## Materials and Methods

### Data Sources

The RNA-seq data of 40 samples from leaf and silique tissues of multi genotypes of *Arabidopsis* in the present study were from our previous study, including the overexpression of *AtLEA*, *AtVOC*, RNAi of *AtVOC*, and *AtLEA* mutant (Liang et al., 2019). Detailed sample information is listed in Table 1. Based on these data, we explored the expression levels of known and newly predicted lncRNA, and the expression patterns of lncRNA in transgenic, overexpression, mutation, and RNAi-treated samples under long-term drought treatment and short-term drought treatment conditions were studied, as well as the regulation of the expression of protein-coding genes.

**TABLE 1:** RNA-seq analysis codes for the drought treatments combining the genotype and sample tissues.

**Code**	**Genotype**	**Drought treatment**	**Sample tissue**
AtLEALL	Over expression of AtLEA	Long-term	Leaves
AtVOCLL	Over expression of AtVOC	Long-term	Leaves
mLEALL	Mutant of AtLEA	Long-term	Leaves
AtVOCRNAiLL	RNAi of AtVOC	Long-term	Leaves
WTLL	Wild type	Long-term	Leaves
AtLEALS	Over expression of AtLEA	Short-term	Leaves
AtVOCLS	Over expression of AtVOC	Short-term	Leaves
mLEALS	Mutant of AtLEA	Short-term	Leaves
AtVOCRNAiLS	RNAi of AtVOC	Short-term	Leaves
WTLS	Wild type	Short-term	Leaves
AtLEASL	Over expression of AtLEA	Long-term	Silique
AtVOCSL	Over expression of AtVOC	Long-term	Silique
mLEASL	Mutant of AtLEA	Long-term	Silique
AtVOCRNAiSL	RNAi of AtVOC	Long-term	Silique
WTSL	Wild type	Long-term	Silique
AtLEASS	Over expression of AtLEA	Short-term	Silique
AtVOCSS	Over expression of AtVOC	Short-term	Silique
mLEASS	Mutant of AtLEA	Short-term	Silique
AtVOCRNAiSS	RNAi of AtVOC	Short-term	Silique
WTSS	Wild type	Short-term	Silique

### Data Processing

After removing sequencing adaptors and low-quality sequences using Cutadapt (Martin, 2011), the RNA-seq clean data of each sample was aligned to the *Arabidopsis* reference genome using HiSAT2 (Kim et al., 2015). RNA-seq alignment results of each sample were further assembled and merged by StringTie (Pertea et al., 2015).

### Identification of lncRNAs

To obtain qualified lncRNA candidates, we performed a preliminary filtering of transcripts and predicted the coding potential of the screened transcripts. Four principles were employed in the filtering step (Jia et al., 2020): (1) Transcript length and exon number should be above 200 bp and 2, respectively. (2) Transcripts should be covered by at least five reads in all samples. (3) Transcripts aligned to known mRNAs and other non-coding RNAs (such as rRNA, tRNA, snoRNA, snRNA) of this species should be discarded. (4) The potential lncRNA, intronic lncRNA, and anti-sense lncRNA were identified according to class_code information (“u,” “i,” “x”). Lacking coding ability, the candidate lncRNA transcripts were further measured on coding potential using four methods. The coding-non-coding index (CNCI) (Sun et al., 2013), coding potential calculator (CPC) (Kong et al., 2007), and coding potential assessment tool (CPAT) (Wang et al., 2013) were utilised to calculate the encoding and non-coding capabilities of transcripts. In addition, the protein domains in HMM library were searched in each candidate sequence using pfamscan^1^ tool to screen out sequences with known protein domains.

### Joint Analysis of lncRNA and mRNA

The DESeq (Anders and Huber, 2010) was used for differential expression analysis, and genes with | log_2_ ratio| ≥ 1 and *q* < 0.05 as a cut-off for significant differential expression were selected. The lncRNA mainly binds to regulatory regions of protein-coding genes and regulates gene expression in *cis* or *trans* formations. For differentially expressed lncRNAs, *cis* and *trans* regulation analyses were performed, and their function was indirectly predicted by the target genes. *Cis* regulation refers to the function of lncRNA that is related to the protein-encoding genes adjacent to it at the genomic locus. Therefore, the prediction method of the target gene for *cis* regulation is to screen out protein-encoding genes adjacent to lncRNA (upstream and downstream 50 Kb) as target genes. The *trans*-regulated genes were recognised according to the correlation coefficient of lncRNA and mRNA expression values (pearson correlation coefficient ≥ 0.9 or pearson correlation coefficient ≤ −0.9). Heatmaps of the differentially expressed lncRNA and mRNAs were plotted with the Complexheatmap package (Gu et al., 2016). The qPCR were performed based by the methods in previous study (Liang et al., 2019).

### Function Analysis of Differentially Expressed lncRNA Target Genes

The target genes were analysed for enriched GO (Harris et al., 2004) and KEGG categories (Kanehisa and Goto, 2000) by applying hypergeometric tests. An adjusted *P*-value of 0.05 was used as a threshold for significant enrichment.

## Results

### The Identification of lncRNAs and Their Expression Patterns

**FIGURE 1:**
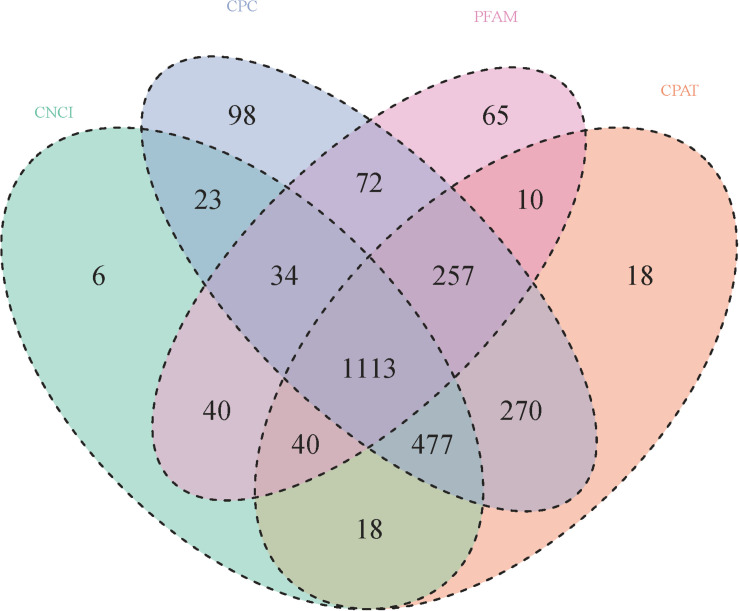
Numbers of novel lncRNAs in 56 samples (details in Table 1). Novel lncRNAs were identified by using CNCI, CPC, and CPAT software, and compared with the pfam database, respectively.

**FIGURE 2:**
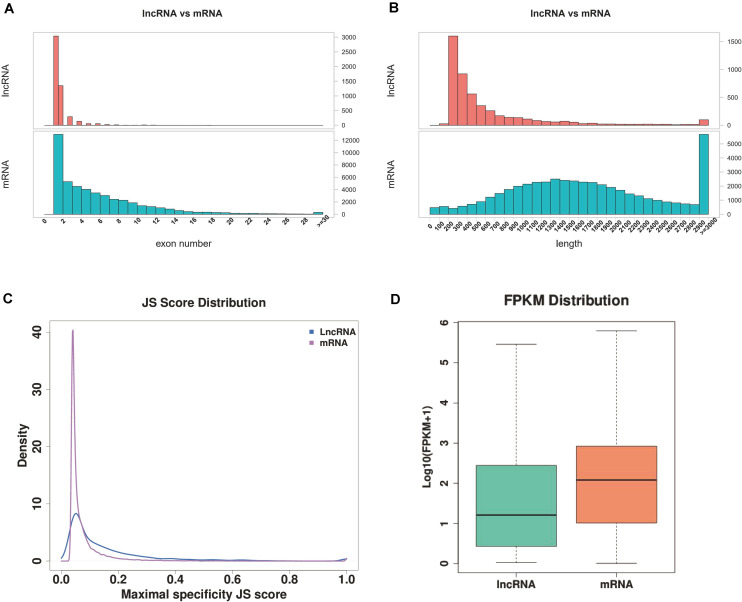
Comparison of IncRNA and mRNA. **(A)** Exon numbers of IncRNA and mRNA. **(B)** Length of IncRNA and mRNA. **(C)** JS score distribution of IncRNA and mRNA. **(D)** FPKM distribution of IncRNA and mRNA.

A total of 2,744 known lncRNAs were identified in 40 samples, 1,751, 2,344, 2,203, and 1,631 novel lncRNAs were identified by using CNCI, CPC, and CPAT software, and pfam database, respectively. Among these lncRNAs, 1113 new lncRNAs were shared in the results of all four methods. Compared to lncRNA, the mRNAs presented a larger exon number and a longer gene length (Figures 1, 2A,B).

The FPKM of transcripts for mRNA and lncRNA in *Arabidopsis* were calculated in order to explore the expression patterns of identified lncRNAs, and a lower expression level of lncRNA (Wilcoxon rank sum test *P* < 0.001) than protein-coding genes were observed (Figure 2D). To quantitatively evaluate the expression specificity of each transcript, an entropy-based metric relying on the Jensen-Shannon (JS) distance was employed to calculate the expression-specific score for each transcript. For each transcript, the maximum JS score of each RNA was considered to be tissue-specific. A large proportion of lncRNA exhibited a higher maximal JS score (Figure 2C), indicating that the identified lncRNA carries a stronger tissue-specificity of expression.

### Expression Level of lncRNA in Siliques and Leaves Under Long-Term Drought Treatment

**FIGURE 3:**
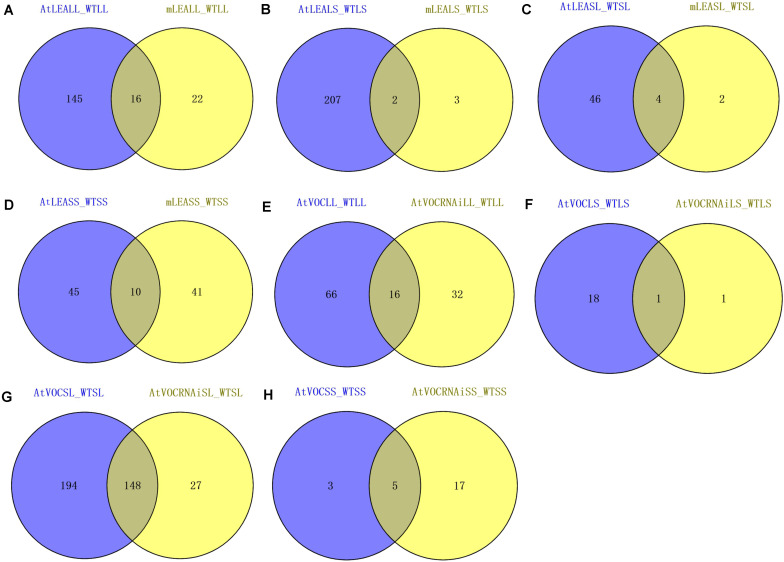
Numbers of differentially expressed lncRNAs in samples. **(A,E)** Leaves samples in long-term drought; **(B,F)** leaves samples in short-term drought; **(C,G)** silique samples in long-term drought; **(D,H)** silique samples in short-term drought. For leaf samples, by using the wild type long-term treatment sample (WTLL) and wild type short-term treatment sample (WTLS) as controls (the first letter “L” represents leaf samples, the second letters “L” and “S” represent long-term and short-term drought treatments, respectively, the first letter “m” represents mutants). For siliques, using WTSL and WTSS (the first letter “S” represents the silique sample, and the second letter “L” and “S” indicate long-term and short-term drought treatments, respectively, the first letter “m” indicates mutants) as controls.

**FIGURE 4:**
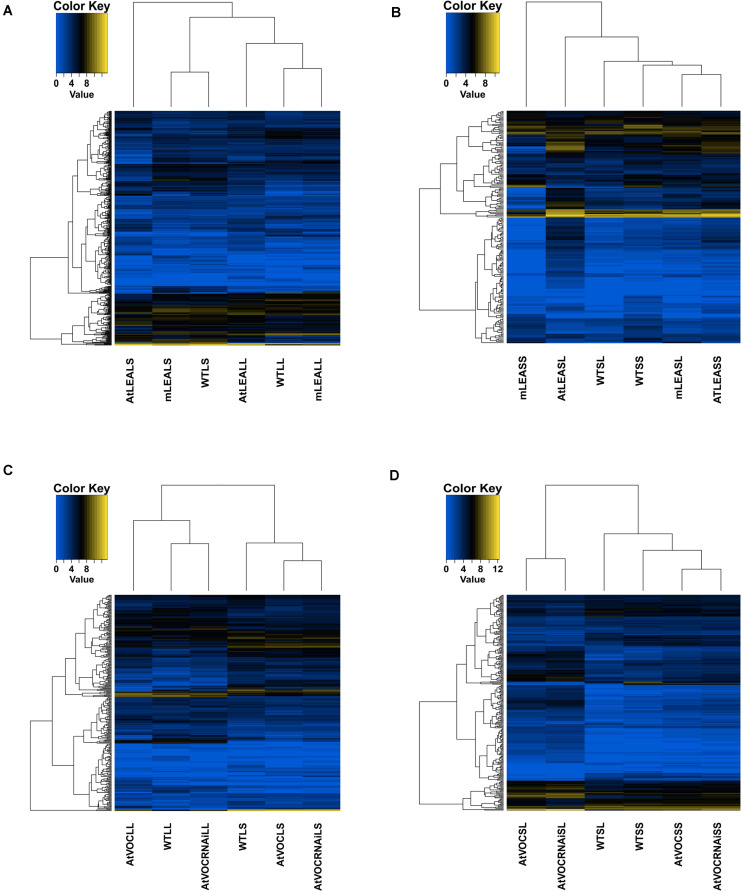
Heatmaps of differentially expressed lncRNAs in samples. **(A)** Leaves of LEA relative samples; **(B)** siliques of LEA relative samples; **(C)** leaves of VOC relative samples; **(D)** siliques of VOC relative samples.

For leaf samples, the RNA sequencing data of AtLEALL, AtVOCLL, AtVOCRNAiLL, mLEALL, AtLEALS, AtVOCLS, AtVOCRNAiLS, and mLEALS were analysed. Compared with WTLS, 209 and 5 differentially expressed lncRNAs in AtLEALS and mLEALS were detected, respectively. Compared with WTLL, 161 and 38 differentially expressed lncRNAs for AtLEALL and mLEALL under long-term treatment conditions were also detected (Figures 3A,B, 4A). The present results indicated that the AtLEA over-expressed lines could induce more changes of lncRNA expression levels than mutant lines under both long-term and short-term drought treatment conditions.

For leaf samples focussing on *VOC*, 19 and 2 differentially expressed lncRNAs were identified from AtVOCLS and AtVOCRNAiLS under short-term drought treatment conditions compared with WTLS, respectively. Of these, only one lncRNA was shared. Under long-term drought treatment, 82 and 48 differentially expressed lncRNAs from AtVOCLL and AtVOCRNAiLL were identified compared with WTLL, respectively. Of which, 16 lncRNAs were shared (Figures 3E,F, 4C). These results indicated that the changes of target genes of lncRNAs that effected by *VOC* was mainly occurred from long-term drought treatment.

For siliques, the sequencing sample data of AtLEASL, AtVOCRNAiSL, AtVOCSL, mLEASL, ATLEASS, AtVOCRNAiSS, AtVOCSS, and mLEASS were further analysed. For the *LEA* gene of siliques under short-term treatment, 55 and 51 differentially expressed lncRNAs in samples ATLEASS and mLEASS were detected compared with WTSS, respectively. 50 and 6 differentially expressed lncRNAs for AtLEASL and mLEASL were detected under long-term treatment conditions (Figures 3C,D, 4B). The results indicated that more differentially expressed lncRNA were produced in the mutant lines of LEA under short-term drought treatments.

In silique samples focussing on the *VOC* gene under short-term treatment 22 and 8 differentially expressed lncRNAs for samples AtVOCRNAiSS and AtVOCSS were detected compared with WTSS, respectively. 175 and 342 differentially expressed lncRNAs in AtVOCRNAiSL and AtVOCSL under long-term treatment conditions were detected (Figures 3G,H, 4D).

### Effects of lncRNA Expression Level on Lipid Metabolism and Photosynthetic Carbon Sequestration

**FIGURE 5:**
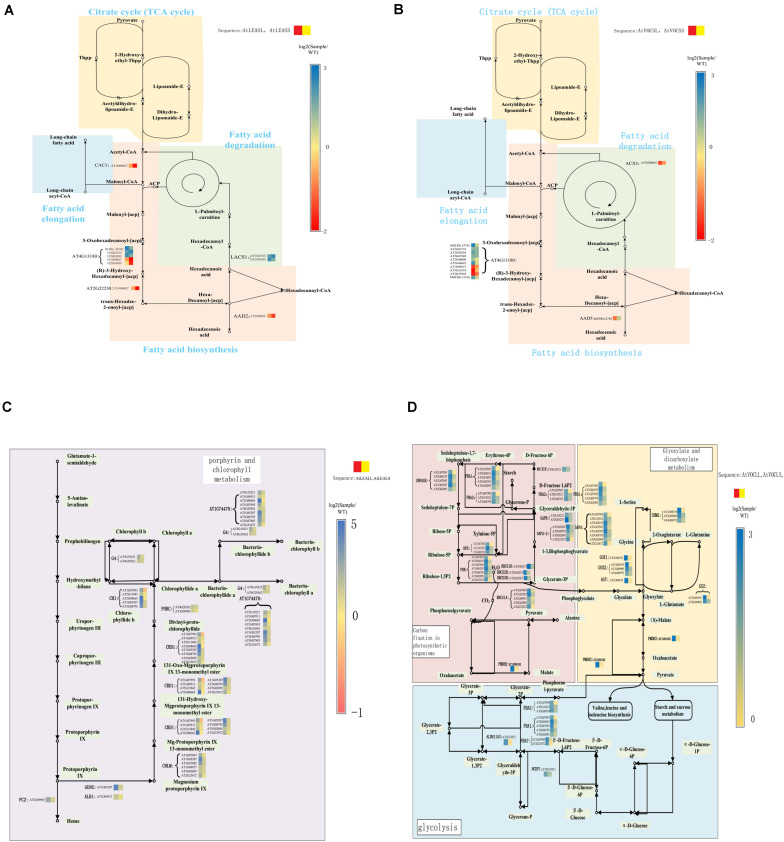
Key genes expression in crucial pathways. **(A,B)** Pathways of FA metabolism; **(C)** porphyrin and chlorophyll metabolism; **(D)** glyoxylate and dicarboxylate metabolism.

It would be enhanced the biosynthesis and attenuated degradation of fatty acid (FA) in the *LEA* overexpression lines as revealed in our previous study (Liang et al., 2019). KEGG pathway analysis showed that a number of key enzyme genes that involved in FA synthesis and degradation pathways were differentially expressed in these *LEA*-overexpressing plants. Interestingly, based on lncRNA target gene prediction results, we also discovered that several genes that differentially expressed in the FA synthesis and degradation pathways were regulated by lncRNAs (Figure 5A). These lncRNAs were also differentially expressed in *LEA*-overexpressing plants. For example, AT4G13180 [NAD (P) -binding Rossmann-fold superfamily protein] was regulated by five differentially expressed lncRNAs.

*VOC* genes were actively involved in FA degradation and FA elongation, and multiple genes related to FA degradation were downregulated in *VOC* over-expressed samples (Liang et al., 2019). The lncRNA analysis revealed that only few FA-related genes were regulated by lncRNA in *VOC* over-expressed samples (Figure 5B). Ten differentially expressed lncRNAs exhibited a regulatory function on AT4G13180. Moreover, the regulation patterns of these lncRNAs on AT4G13180 were not consistent. The lncRNAs AT1G09627, AT3G24518, and AT2G35945 were downregulated in AtVOCSL, and lncRNA MSTRG.35793 was upregulated in AtVOCSL.

The KEGG pathway analysis of the lncRNA target genes also showed that the genes involved in carbon fixation and chlorophyll metabolism of photosynthetic organisms could be regulated by multiple lncRNAs. Most of the lncRNAs that regulate these genes were upregulated in *LEA*-overexpressing samples (Figure 5C). However, the expression level of lncRNAs in samples did not exhibit a significant difference under short-term drought conditions. In long-term drought conditions, lncRNAs in AtLEA overexpressing samples tended to play a regulatory role (Figure 5C) to assist the plants to obtain a stronger photosynthetic capacity and thus stronger drought resistance.

Metabolism of glyoxylic acid and dicarboxylic acid as well as plant hormone signal transduction, was vigorous in the leaves of transgenic rapeseed *VOC* gene overexpression samples. Additionally, the results of lncRNA target genes analysis indicated that the extensively downregulated lncRNAs were involved in the regulation of genes involved in the metabolism of glyoxylate, dicarboxylic acid, and transduction of plant hormone signals (Figure 5D). In the short-term drought treatment, *AGT*, *GOX1*, *GOX2*, and *PMDH2* genes were regulated by several upregulated lncRNAs, which further revealed that glyoxylate metabolism and oxygen-related metabolic processes were affected by *VOC* genes (Figure 5D).

### GO Enrichment of Target Genes of Differentially Expressed lncRNAs

**FIGURE 6:**
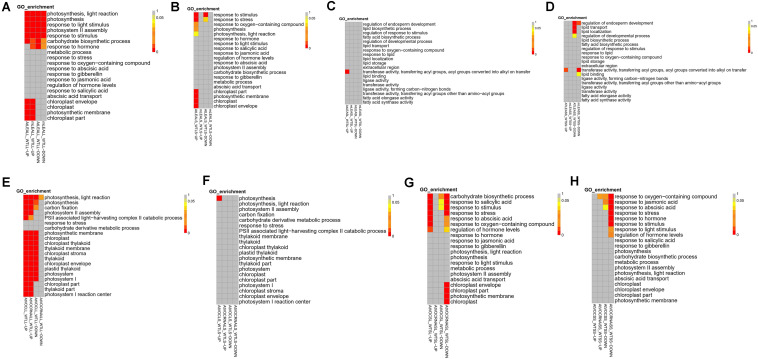
GO enrichment of differentially expressed lncRNAs in samples. **(A,E)** Leaves samples in long-term drought; **(B,F)** leaves samples in short-term drought; **(C,G)** silique samples in long-term drought; **(D,H)** silique samples in short-term drought.

GO analysis was performed on the target genes of differentially expressed lncRNAs. In the silique of *LEA*-overexpressing plants, genes regulated by upregulated lncRNA under short-term or long-term drought treatment were significantly enriched in transferase activity, transferring acyl groups, and acyl groups converted into alkyl groups on transfer (Figures 6C,D). Moreover, under short-term drought treatment, downregulated lncRNA also affected the genes that related to the regulation of endosperm development, lipid transport, and lipid localisation in *LEA*-overexpressing plants. In contrast, lncRNA of the mutant plants under long-term treatment did not exhibit a significant effect on the functions of these genes, but under short-term treatment, a downregulating effect on genes with functions such as regulation of endosperm development was observed.

In leaves, overexpression of *LEA* affected multiple genes that related to photosynthesis and stress resistance (Figures 6A,B). Under short-term and long-term treatment conditions, the target genes of up-regulated lncRNA in the chloroplast envelope, chloroplast, photosynthetic membrane and chloroplast were enriched. Moreover, additional functions such as response to stress and oxygen-containing compounds were also regulated by lncRNA during short-term treatment. These results suggested that a number of genes related to stress resistance are rapidly regulated by lncRNA in *Arabidopsis* in early stage of treatment under the condition of *LEA* overexpression, while stress responses that are based on the lncRNA regulation are fewer in the mutant plants under short-term drought treatment.

Compared with *LEA*, the overexpression of *VOC* genes induced more lncRNAs in leaves (especially upregulated lncRNA) to participate in the regulation of photosynthesis and stress-related genes (Figures 6E,F). Under short-term treatment, the target genes in AtVOC over-expressing plants were mainly participated in the regulation of photosynthesis. Notably, in AtVOCRNAi plants, short-term treatment did not stimulate the regulation of stress and photosynthesis by lncRNA, but was consistent with AtVOCLS under long-term conditions (Figures 6G,H).

AtVOC over-expressing plants produced upregulated lncRNA mostly under long-term drought treatment, which significantly regulated the carbohydrate biosynthetic process, response to salicylic acid, and other functions. For AtVOCRNAi-treated plants, regardless of short-term or long-term drought treatment, down-regulated lncRNA were produced in response to some of the above functions, indicating that the suppression of the *VOC* gene had a negative effect on short-term stress and long-term adaptation of plants, which was not conducive to plant resistance to stress.

### Specific Expression Pattern of lncRNA Trans-Regulatory Target Genes

**FIGURE 7:**
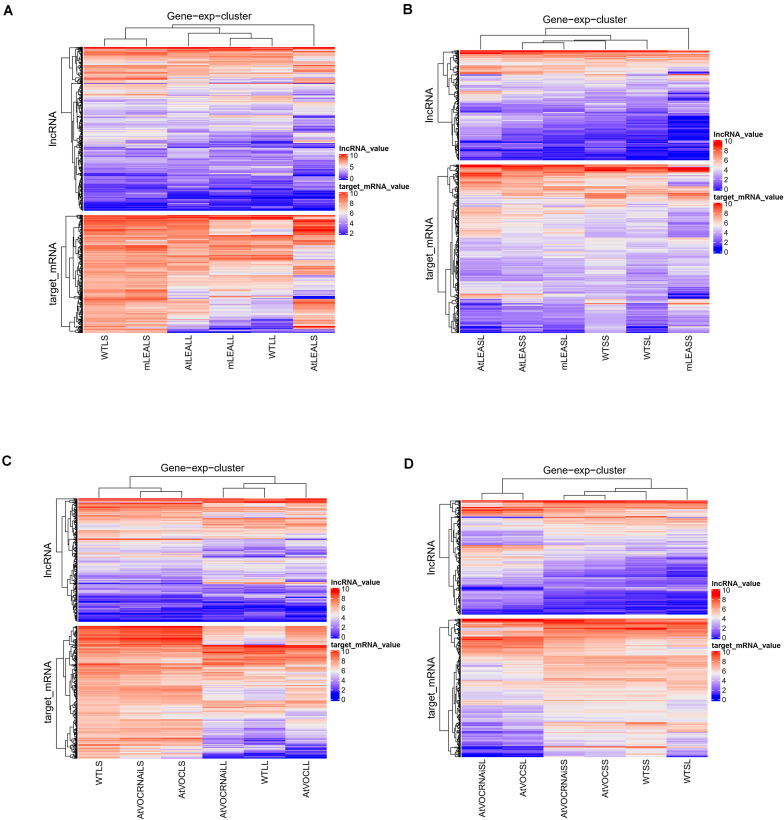
Heatmaps of IncRNAs and target mRNAs. **(A)** Leaves of LEA relative samples; **(B)** siliques of LEA relative samples; **(C)** leaves of VOC relative samples; **(D)** siliques of VOC relative samples.

The trans-regulatory target genes of the differentially expressed lncRNAs were predicted by the absolute values of correlation coefficient between lncRNA and mRNA expression values. A heatmap was used to show the expression pattern of differentially expressed lncRNAs and its target genes (Figure 7). The figure indicates that the expression pattern of these target genes were line-specific. The trans-regulatory target genes were almost all high expressed in WTLS, mLEALS, AtVOCRNAiLS, and WTSS (Figures 7A,C,D). Meanwhile, as Figure 7 shows, the expression level of lncRNAs were lower than its trans-regulatory target genes.

## Discussion

The stomata of *LEA* and *VOC*-OE transgenetic lines were regularly closed under drought conditions, which could maintain their normal shape and function, while the leaf surface of the wide type were possessed more wrinkles and tended to dry up under drought stress. Previous studies have shown that a number of drought marker genes, including *RD29A*, *SnRK2.6*, and *RbohD*, were highly expressed in the OE line (Liang et al., 2019), However, it was mainly focussed on the changes in the expression of coding genes and essential pathways, such as ABA signalling and photosynthesis pathways. In the present study, we focussed on the changes of lncRNA expression and the effects of these differentially expressed lncRNAs on the regulation of coding genes against drought. The previous study showed that *VOC* genes were able to affect late silique development under drought conditions (Liang et al., 2019). The present results further suggest that lncRNA is more likely to participate in the regulation of *VOC* genes during late silique development. At the same time, the present results shown that compared to the lncRNA, the mRNAs had a larger exon number and a longer gene length (Figures 1, 2A,B), which concurs with a previous study (Lagarde et al., 2017). An expression difference between mRNA and lncRNA is also consistent with previous reports (Deng et al., 2018). The identified lncRNA carries a stronger tissue-specificity of expression (Figure 2C), which is consistent with previous findings that the expression pattern is heterogeneous between lncRNA and mRNA (Chen et al., 2018).

Studies on various plants, such as rice and cotton have shown that lncRNAs are essential to the resistance of plants to drought stress (Chen et al., 2018; Deng et al., 2018). Predicting the function of lncRNA target genes could accelerate the study of lncRNAs. The predicated possible target genes of differentially expressed lncRNAs revealed that some target genes were significantly enriched in different pathways. Photosynthesis is fundamental in the synthesis of carbohydrates, which is a critical raw material for plants to further synthesise fatty acids. The photosynthesis-related genes analysis results indicated that the carbon fixation and chlorophyll metabolism of photosynthetic organisms were upregulated in *LEA* overexpression lines, and *LEA* was able to activate plants to improve photosynthesis capacity under drought stress (Liang et al., 2019). Furthermore, under adverse environments, lncRNA participates in regulating photosynthetic pathways, which are essential for plants to resist stress (Scherer et al., 1984; Colom and Vazzana, 2003; Xu et al., 2019; Zeng et al., 2019). When the genes are far away from lncRNAs, regulation occurs through trans-action, and then the expression levels of target genes were changed. These trans-action lncRNAs can guide ribonucleoprotein (RNP) complexes to specific genome locations or recruit chromatin-modifying enzymes to target genes (Wu et al., 2020). This study revealed that, a large number of up-regulated lncRNAs were involved in trans-regulation of target genes involved in photosynthesis and chlorophyll synthesis pathways in *LEA*-overexpressing plants, among which, some genes were trans-regulated by multiple lncRNAs. *CRD1* and *CHLM* are essential genes in the process of chlorophyll synthesis (Kobayashi and Masuda, 2016; Herbst et al., 2018; Zubo et al., 2018), the present study revealed that the *CRD1* was trans-regulated by eight lncRNAs including AT1G07993, AT1G09513, AT5G15845, AT2G08665, AT1G05207, AT3G08795, AT5G02095, and AT2G23672; and the *CHLM* was trans-regulated by six lncRNAs including AT1G07693, AT1G05207, AT5G07885, AT1G09927, AT5G02095, and AT2G23672. In *Cucumis*, the *CHLM* was regulated by LINC-chc01G00070-1 when the plant response to allopolyploidization, indicating that *CHLM* is involved in the lncRNA-mediated regulatory mechanisms (Wang et al., 2020). The fact that a large number of upregulated lncRNAs were involved in the regulation of these genes, further reveals that lncRNA regulation of the photosynthesis and chlorophyll synthesis pathways is a key factor in trigging the stress response in plants. Pearson correlation analysis also showed that these lncRNAs were positively correlated with *CRD1* and *CHLM*. In addition, *CRD1* and *CHLM* were significantly upregulated in *LEA*-overexpressing lines. The maintenance of chlorophyll synthesis in leaves may assist plants to enhance photosynthesis in arid environments, thereby further promoting the accumulation of FA. The lncRNA in *LEA*-OE and *VOC*-OE strains exhibited a positive regulatory effect on photosynthetic efficiency, which could further enhance the drought resistance and promote lipid accumulation.

In the leaves of *VOC-*overexpressing plants, lncRNA also showed very interesting results in the regulation of methylglyoxal (MG). MG is a highly cytotoxic and mutagenic compound. Under normal growth conditions, the basal level of MG in plants is low, and it acts as an important signalling molecule. However, when the plant is subjected to abiotic stress, MG can accumulate, and acts as a toxic molecule under this situations, inhibiting different developmental processes, including seed germination, photosynthesis, and root growth (Hoque et al., 2016; Li et al., 2017), a large number of down-regulated lncRNAs in *VOC*-OE were involved in the regulation of the MG synthesis pathway were observed. The key genes, including *SHM1*, *GOX2*, and *GS2*, involved in the methylglyoxal synthesis pathway, were all regulated by multiple down-regulated lncRNAs, which were positively correlated with the expression levels of *SHM1*, *GOX2*, and *GS2*. Through such regulation, the accumulation of MG was reduced. In another study, the *GOX2* was trans-regulated by lncRNA MSTRG.14375 under infection of downy mildew in Chinese cabbage (Zhang et al., 2021). That study shows that *GOX2* is the target of lncRNA regulation in response to stress. These results indicated that when *VOC* is overexpressed in *Arabidopsis*, lncRNAs produced under drought stress were able to regulate the glyoxylate enzyme system to a certain extent, and thus could be a factor in the response to drought stress.

Previous studies (Liang et al., 2019) have demonstrated that the oil content of *LEA*-OE and *VOC*-OE lines has increased. Under drought stress conditions, *LEA*-OE would drive seeds to form a stable and thick cell membrane to protect lipid synthesis in plants. In the siliques of the *VOC* and *LEA* overexpression lines, lncRNAs were not intensively involved in the regulation of the fatty acid synthesis pathway. However, but a few genes were still regulated by multiple differentially expressed lncRNAs, for example in *LEA*-OE, AT4G13180 was regulated by seven lncRNAs, of which five were upregulated, both under long-term and short-term drought treatments, and this promoted fatty acid synthesis.

## Conclusion

In summary, the changes in lncRNA caused by the expression of *LEA* and *VOC* genes may be related to the drought tolerance and oil content of *Arabidopsis thaliana*, and the lines that overexpressed these two genes showed strong drought resistance and oil production. *LEA* and *VOC* increase photosynthetic efficiency and reduce reactive oxygen species under drought stress. For future breeding purposes, one of the regulatory methods to achieve this goal is to regulate the key genes involved in these two pathways through up-regulation and down-regulation of the related lncRNAs. Consequently, these drought-responsive lncRNAs not only played a role in the drought stress, but also promote oil accumulation, which requires further investigation. In short, this study provides a novel perspective for the study of the relationship between oil accumulation and drought resistance from the regulatory relationship between lncRNA and mRNA, offering a new approach to further promote the breeding of oil crops.

## Data Availability Statement

The original contributions presented in the study are included in the article/Supplementary Material, further inquiries can be directed to the corresponding author/s.

## Author Contributions

ML designed the experiments. YH and KC conducted the bioinformation analysis. CL performed the data collection. YL wrote the manuscript. All authors contributed to the article and approved the submitted version.

## Conflict of Interest

The authors declare that the research was conducted in the absence of any commercial or financial relationships that could be construed as a potential conflict of interest.

## Footnotes

1 https://www.ebi.ac.uk/Tools/pfa/pfamscan/

## Supplementary Material

Supplementary Figure 1 The qPCR results of some lines and time points, and it was compared with the RNA-seq data.

Supplementary Table 1 SRA accession number of RNA-seq data uploaded to the NCBI database.

Supplementary Table 2 LncRNAs and their trans-regulatory target genes in leaves of LEA relative samples.

Supplementary Table 3 LncRNAs and their trans-regulatory target genes in siliques of LEA relative samples.

Supplementary Table 4 LncRNAs and their trans-regulatory target genes in leaves of VOC relative samples.

Supplementary Table 5 LncRNAs and their trans-regulatory target genes in siliques of VOC relative samples.

Supplementary Table 6 Differentially expressed lncRNAs between AtLEALL and WTLL samples.

Supplementary Table 7 Differentially expressed lncRNAs between AtLEALS and WTLS samples.

Supplementary Table 8 Differentially expressed lncRNAs between AtLEASL and WTSL samples.

Supplementary Table 9 Differentially expressed lncRNAs between AtLEASS and WTSS samples.

Supplementary Table 10 Differentially expressed lncRNAs between AtVOCLL and WTLL samples.

Supplementary Table 11 Differentially expressed lncRNAs between AtVOCLS and WTLS samples.

Supplementary Table 12 Differentially expressed lncRNAs between AtVOCSL and WTSL samples.

Supplementary Table 13 Differentially expressed lncRNAs between AtVOCSS and WTSS samples.

Supplementary Table 14 Primers used in qPCR.

## Abbreviations

lncRNAs: Long non-coding RNAs
OE: over-expressed
DREBs: dehydration response element binding proteins
CNCI: coding-non-coding index
CPC: coding potential calculator
CPAT: coding potential assessment tool
JS: Jensen-Shannon
WTLL: wild type long-term treatment samples
WTLS: wild type short-term treatment samples.
