# Supplementary material for: Transcriptome Profile Analysis of Arabidopsis Reveals the Drought Stress-Induced Long Non-coding RNAs Associated With Photosynthesis, Chlorophyll Synthesis, Fatty Acid Synthesis and Degradation
Source: Front Plant Sci. 2021 May 25;12:643182. doi: 10.3389/fpls.2021.643182 (PMC8185149; doi:10.3389/fpls.2021.643182)
Supplement: Supplementary Figure 1 — The qPCR results of some lines and time points, and it was compared with the RNA-seq data. [file Image_1.PDF]

q-RTPCR

		mLEA	BnLEA-35S	AtLEA-35S	AtVOC-RNAi	BnVOC-35S	AtVOC-35S
Lipase	AT5G40990		0.07	0.01	/	0.00	/
nata1	AT2G39030	-5.03	-7.78	-2.87	7.64	-18.64	-6.00
B3	AT4G05630	0.18	-0.11	/	0.00	12.05	2.18
ASK16	AT2G03190	-0.10	5.95	5.24	19.93	7.26	3.58
P450	AT1G65670	/	-0.05	0.02	-1.40	-6.35	-8.27
WRKY62	AT5G01900	/	/	0.01	-0.02	12.84	/
MAPKKK21	AT4G36950	0.01	0.07	0.08	1.83	-0.07	3.54
CAB3	AT1G29910	0.07	-1.04	/	/	/	0.50
PSAN	AT5G64040	-0.06	0.07	-0.41	/	/	0.70
BHLH38	AT3G56970	/	/	0.08	/	/	/
DREB2C	AT2G40340	/	1.12	0.02	/	0.05	-0.07
HSP20	AT1G07400	/	-0.14	/	/	-0.04	0.00
FAD8	AT5G05580	/	-0.17	0.02	0.00	0.08	/
LOX2	AT3G45140	/	-0.06	/	-7.89	/	-0.92

RNA-Seq

		mLEA	BnLEA-35S	AtLEA-35S	AtVOC-RNAi	BnVOC-35S	AtVOC-35S
Lipase	AT5G40990	/	/	/	/	/	/
nata1	AT2G39030	-3.12	-8.8042	-5.9433	-10.3	-5.5365	-12.28
B3	AT4G05630	/	/	/	/	4.1743	3.067
ASK16	AT2G03190	/	4.4833	3.133	4.8626	5.24	4.3951
P450	AT1G65670	/	/	/	-2.5912	-7.1256	-1.759
WRKY62	AT5G01900	/	/	/	/	5.62	/
MAPKKK21	AT4G36950	/	/	/	1.5886	/	1.8472
CAB3	AT1G29910	/	/	/	/	/	/
PSAN	AT5G64040	/	/	/	/	/	/
BHLH38	AT3G56970	/	/	/	/	/	/
DREB2C	AT2G40340	/	/	/	/	/	/
HSP20	AT1G07400	/	/	/	/	/	/
FAD8	AT5G05580	/	/	/	/	/	/
LOX2	AT3G45140	/	/	/	-2.6749	/	-3.5704

q-RTPCR

		mLEA-D	BnLEA-35S-D	AtLEA-35S-D	AtVOC-RNAi-D	BnVOC-35S-D	AtVOC-35S-D
Lipase	AT5G40990	/	/	0.01	0.47	0.13	0.38
nata1	AT2G39030	-0.04	-0.83	-1.17	-1.01	-5.01	-0.94
B3	AT4G05630	0.05	/	/	/	/	/
ASK16	AT2G03190	/	/	/	0.38	0.01	/
P450	AT1G65670	-0.22	-0.31	-1.14	-0.15	0.00	-1.03
WRKY62	AT5G01900	-0.05	/	0.06	0.00	0.03	/
MAPKKK21	AT4G36950	0.00	0.02	/	0.02	-0.09	0.00
CAB3	AT1G29910	-0.01	1.40	2.42	0.07	/	/
PSAN	AT5G64040	/	0.32	/	-0.02	/	/
BHLH38	AT3G56970	/	0.04	0.00	/	0.03	/
DREB2C	AT2G40340	/	/	/	/	2.06	/
HSP20	AT1G07400	3.41	/	-0.06	0.01	0.83	1.13
FAD8	AT5G05580	/	0.09	/	/	0.00	0.06
LOX2	AT3G45140	-1.99	-2.43	-2.71	/	/	-0.65

RNA-Seq

		mLEA-D	BnLEA-35S-D	AtLEA-35S-D	AtVOC-RNAi-D	BnVOC-35S-D	AtVOC-35S-D
Lipase	AT5G40990	/	/	/	/	/	/
nata1	AT2G39030	/	-4.2973	-4.0966	-2.5291	-1.9582	-1.774
B3	AT4G05630	/	/	/	/	/	/
ASK16	AT2G03190	/	/	/	/	/	/
P450	AT1G65670	-1.1417	-1.7437	-2.0755	-2.2437	/	-2.0925
WRKY62	AT5G01900	/	/	/	/	/	/
MAPKKK21	AT4G36950	/	/	/	/	/	/
CAB3	AT1G29910	/	0.62915	0.61363	/	/	/
PSAN	AT5G64040	/	0.53947	/	/	/	/
BHLH38	AT3G56970	/	/	/	/	/	/
DREB2C	AT2G40340	/	/	/	/	2.3101	/
HSP20	AT1G07400	1.5759	/	/	/	1.4085	1.3524
FAD8	AT5G05580	/	/	/	/	/	/
LOX2	AT3G45140	-0.59502	-1.1339	-1.3249	/	/	-0.95928

q-RTPCR

		mLEALD	BnLEALD	AtLEALD	AtVOC-RNAiLD	BnVOCLD	AtVOCLD
Lipase	AT5G40990	0.10	-3.03	-2.30	-2.50	/	0.01
nata1	AT2G39030	/	-1.94	-6.59	0.03	-4.91	/
B3	AT4G05630	/	0.08	/	0.00	/	/
ASK16	AT2G03190	/	/	0.06	/	0.09	/
P450	AT1G65670	0.04	0.06	0.02	-0.09	/	/
WRKY62	AT5G01900	0.02	2.19	1.38	/	-0.08	5.18
MAPKKK21	AT4G36950	/	0.01	-0.82	/	/	0.01
CAB3	AT1G29910	0.04	/	0.36	0.09	/	0.03
PSAN	AT5G64040	0.00	/	/	0.09	/	/
BHLH38	AT3G56970	/	2.40	/	0.09	5.92	/
DREB2C	AT2G40340	0.01	0.01	-1.39	/	-2.14	/
HSP20	AT1G07400	-0.07	-0.01	-10.02	/	-10.01	-0.01
FAD8	AT5G05580	/	3.78	1.93	0.04	2.52	/
LOX2	AT3G45140	/	1.27	/	/	0.00	0.01

RNA-Seq

		mLEALD	BnLEALD	AtLEALD	AtVOC-RNAiLD	BnVOCLD	AtVOCLD
Lipase	AT5G40990	/	-3.4821	-1.8923	-3.9016	/	/
nata1	AT2G39030	/	-2.722	-3.997	/	1.0492	/
B3	AT4G05630	/	/	/	/	/	/
ASK16	AT2G03190	/	/	/	/	/	/
P450	AT1G65670	/	/	/	/	/	/
WRKY62	AT5G01900	/	4.7127	4.1432	/	/	3.3042
MAPKKK21	AT4G36950	/	/	/	/	/	/
CAB3	AT1G29910	/	/	/	/	/	/
PSAN	AT5G64040	/	/	/	/	/	/
BHLH38	AT3G56970	/	3.2868	/	/	5.02	/
DREB2C	AT2G40340	/	/	-4.1001	/	-4.8519	/
HSP20	AT1G07400	/	/	-5.1297	/	-5.2462	/
FAD8	AT5G05580	/	2.6936	2.7339	/	2.7552	/
LOX2	AT3G45140	/	1.6865	/	/	/	/

q-RTPCR

		mLEALL	BnLEALL	AtLEALL	AtVOC-RNAiLL	BnVOCLL	AtVOCLL
Lipase	AT5G40990	0.06	/	/	/	/	/
nata1	AT2G39030	0.07	-3.01	-2.14	0.04	0.01	0.01
B3	AT4G05630	/	/	/	/	/	0.00
ASK16	AT2G03190	0.00	/	/	0.00	0.02	/
P450	AT1G65670	0.00	/	/	-0.02	-0.07	0.00
WRKY62	AT5G01900	0.00	-1.08	-1.25	/	0.02	0.00
MAPKKK21	AT4G36950	-0.06	-2.10	/	0.00	-3.02	/
CAB3	AT1G29910	0.02	5.83	10.72	1.98	7.93	/
PSAN	AT5G64040	0.01	1.34	4.46	3.35	/	/
BHLH38	AT3G56970	/	0.01	4.46	/	/	0.00
DREB2C	AT2G40340	0.00	/	0.00	0.00	-1.33	/
HSP20	AT1G07400	-0.09	0.09	0.03	/	/	/
FAD8	AT5G05580	0.07	1.08	/	-0.03	-0.01	0.02
LOX2	AT3G45140	0.00	-1.04	-2.13	-2.37	-2.13	0.01

RNA-Seq

		mLEALL	BnLEALL	AtLEALL	AtVOC-RNAiLL	BnVOCLL	AtVOCLL
Lipase	AT5G40990	/	/	/	/	/	/
nata1	AT2G39030	/	-3.3349	-3.1887	/	/	/
B3	AT4G05630	/	/	/	/	/	/
ASK16	AT2G03190	/	/	/	/	/	/
P450	AT1G65670	/	/	/	/	/	/
WRKY62	AT5G01900	/	-3.0693	-1.6362	/	/	/
MAPKKK21	AT4G36950	/	-3.0021	/	/	-2.9366	/
CAB3	AT1G29910	/	6.13	6.31	2.8728	5.28	/
PSAN	AT5G64040	/	3.8392	3.6074	1.1026	/	/
BHLH38	AT3G56970	/	/	4.2037	/	/	/
DREB2C	AT2G40340	/	/	/	/	-1.2667	/
HSP20	AT1G07400	/	/	/	/	/	/
FAD8	AT5G05580	/	2.0018	/	/	/	/
LOX2	AT3G45140	/	-2.2422	-2.1744	-1.5261	-2.717	/
